# Research Progress in Plant Molecular Systematics of Lauraceae

**DOI:** 10.3390/biology10050391

**Published:** 2021-05-01

**Authors:** Yongjing Tian, Jingbo Zhou, Yunyan Zhang, Shuang Wang, Ying Wang, Hong Liu, Zhongsheng Wang

**Affiliations:** 1 School of Life Sciences, Nanjing University, Nanjing 210023, China; mg1830075@smail.nju.edu.cn (Y.T.); mg1830084@smail.nju.edu.cn (J.Z.); zyynju@smail.nju.edu.cn (Y.Z.); mg20300068@smail.nju.edu.cn (S.W.); wy18851197919@163.com (Y.W.); 2 Center for Tropical Plant Conservation, Fairchild Tropical Botanic Garden, Coral Gables, Miami, FL 33156, USA; 3 Department of Earth and Environment, Florida International University, Miami, FL 33199, USA

**Keywords:** Lauraceae, Laureae, Cinnamomeae, Perseeae, molecular phylogenies, inflorescence

## Abstract

**Simple Summary:**

Lauraceae, as an angiosperm group with high ecological and economic value, has been widely studied. With the development of science and technology, the research of Lauraceae has changed from morphology to molecular systematics. Our paper reviewed the molecular phylogeny of Lauraceae in recent years. From the aspects of gene fragments, chloroplast genome and DNA barcodes, we mainly discussed the establishment of Cinnamomeae in the ‘Core Lauraceae’ and the tribal controversial genera (*Actinodaphne, Sassafras, Caryodaphnopsis, Neocinnamomum* and *Cassytha*). We think that the whole genome and inflorescence characteristics are the breakthrough to solve the tribal problem of Lauraceae. Using reliable molecular and morphological evidence to reconstruct the phylogenetic relationship of Lauraceae will provide an important theoretical basis for the rational utilization of Lauraceae resources, the development of potential resources and the protection of rare plants.

**Abstract:**

Lauraceae is a large family of woody plants with high ecological and economic value. The tribal and generic division and phylogenetic relationship of Lauraceae have long been controversial. Based on morphological and molecular evidence, phylogenetic relationships within the Cinnamomeae, Laureae and Perseeae tribes, also called ‘the Core Lauraceae’, have arisen particular attention. In this review, we comprehensively collated the literatures on the phylogeny of Lauraceae published in recent years and summarized progress made in molecular systematic researches employing gene fragments, chloroplast genomes and DNA barcodings analyses. We clarified the phylogenetic relationships and main controversies of ‘the Core Lauraceae’, the systemic position of fuzzy genera (*Neocinnamomum*, *Caryodaphnopsis* and *Cassytha*) and the development of chloroplast genome and DNA barcodes. We further suggested and proposed the whole genome analysis and different inflorescence types would be possible to provide more information for further research on phylogenetic relationships and taxonomy of Lauraceae.

## 1. Introduction

Lauraceae is a large family of woody plants (with the exception of the genus *Cassytha* being herbaceous), composed of 50 genera and 2500–3000 species [1,2]. The distribution center of modern Lauraceae is tropical to subtropical areas, including Southeast Asia, Mediterranean, Central and South America [3,4,5]. The family has another center of taxonomic diversity in Madagascar [6]. Interestingly, members of Lauraceae are predominant components in evergreen broad-leaved forests in the Old World tropics and subtropics. Meanwhile, they represent a crucial group of moist forests in the New World [7,8,9,10]. Moreover, Lauraceae has high economic value and is an important source of materials on construction, cosmetic, pharmaceutical and other industries [11,12]. For example, *Persea americana* Mill. as a popular fruit tree is now widely planted in tropical regions of the world [13]. *Litsea cubeba* Lour. is an important species for producing essential oils are widely used in perfumes, cosmetics and medicines all over the world [14,15,16]. *Cinnamomum kanehirae*’ s wood has high value because of their massive trunk diameters and aromatic, decay-resistant quality [17].

The origin of Lauraceae can be traced back to the Middle and Late Cretaceous [18,19,20], while the systematic classification of Lauraceae has long been controversial [2,21]. The original Carl von Linné classification [22] contained only two genera, *Laurus* and *Cassytha.* Subsequently, Meissner [23], Bentham & Hooker [24], Pax [25], Mez [26], Kostermans [3], Hutchinson [27], van der Werff [7], van der Werff & Richter [2] and Rohwer [28] established different tribal and generic classification systems within the family. Among them, the classification systems proposed by Kostermans [3] and van der Werff & Richter [2] are considered to be the most relevant [29]. Indeed, these two systems represent the main taxonomic framework of current Lauraceae phylogeny researches [1,9,30,31] (Table 1).

Kostermans [3] classified Lauraceae into two subfamilies, Lauroideae and Cassythoideae. Subfamily Lauriodea includes the following five tribes: Litseeae, Perseeae, Cinnamoneae, Cryptocaryeae and Hypodaphnideae. The establishment of the tribe Cinnamoneae and placement of *Sassafras* in the tribe Cinnamoneae represented the transition from a traditional to a phylogeny-based classification [1,2,9,21,30]. At the proof of chemosystematics of Lauraceae, Gottlieb [32] used the chemical components (including arylpropanoids, alkaloids, flavanoids and terpene) as taxonomic markers to classify Lauraceae tribes, which also supported the tribal division of the taxonomy system put forward by Kostermans [3]. Among subfam. Lauroideae (woody species), tribe Cinnamomeae contained arylpropanoids, Perseeae had simple benzyltetrahydroisoquinoline alkaloids, Litseeae had sesquiterpene chemistry and complex flavanoids, while Cryptocaryeae had various alkaloidals, and subfam. Cassythoideae (the only herbaceous group) had no arylpropanoids’ existences.

Van der Werff and Richter [2] divided Lauraceae into three tribes, i.e., Laureae, Perseae and Cryptocaryeae, based on wood and bark anatomical structure and inflorescence traits. Tribe Cinnamoneae established by Kostermans [3] was dismantled, and the genera *Sassafras* and *Actinodaphne* were merged into Laureae. Van der Werff and Richter [2] integrated traditional macro-morphological traits, mostly from flowers and fruits, with wood and bark anatomical features. They constructed a new Lauraceae classification system including the biogeographic distribution pattern as another significant proof (especially historical distribution records), which has received widespread attention [33].

Li and Li [21] reviewed global researches on the systematic classification of Lauraceae and summarized the researches on plant morphology, palynology, embryology, leaf cuticle, biogeography and molecular systematic. They concluded that the utility of multi-disciplinary approaches to seek evidence, particularly from molecular data, is crucial to solving the systematic problems of Lauraceae. They also emphasized the importance of inflorescence characters in systematics of Lauraceae.

In the earlier molecular phylogeny studies of Lauraceae, the phylogenetic trees constructed by Rohwer [30], Chanderbali et al. [1] and Rohwer & Rudolph [29] all supported a terminal clade that included *Persea* group and the tribes Laureae and Cinnamoneae. However, the resolution of the clade was low, and it was difficult to find morphological synapomorphy to illuminate the explicit phylogenetic relationship of this clade. This terminal monophyletic group was so-called as ‘the Core Lauraceae’. In most studies, their system position is in a state of ambiguity. The other genera’s systematic position and classification have been basically clear, such as *Beilschmiedia* and *Cryptocarya*.

Therefore, this review focuses on the main controversial issues of the phylogenetic research of Lauraceae, including the establishment and composition of three tribes from ‘the Core Lauraceae’, i.e., Cinnamoneae [3], Laureae and Perseeae [2], the systematic relationships among the tribes, and the placement of some representative genera among them. We also summarize the progress on phylogenetic researches that employed chloroplast genome and DNA barcodings data. Our aim is to provide an up-to-date overview of Lauraceae phylogenetic research and its future perspectives.

## 2. The Main Controversy over Cinnamoneae and Laureae Tribes

The tribal placement of *Actinodaphne* and *Sassafras* has been a major controversial issue in the phylogenetic study of the Cinnamoneae and Laureae [34,35,36]. Kostermans [3] placed the genus *Actinodaphne* into the tribe Cinnamoneae based on the proof of phenotypic characteristics: the presence of paniculate inflorescence, no obvious involucrate and the fruit base located in perianth tube. Van der Werff & Richter [2] set this genus in the tribe Laureae on the basis of umbellate inflorescence with decussate bracts, i.e., racemose, shortened inflorescence axis. Nees [37] treated *Actinodaphne* into the tribe Laureae based on inflorescence characteristics.

According to the evidence of molecular phylogenetic researches in recent years, most taxonomists supported the inclusion of *Actinodaphne* in Laureae. For instance, Li et al. [31] constructed a phylogenetic tree for the Lauraceae using the universal cpDNA gene *mat*K and DNA internal transcribed spacer sequences (ITS) and the results showed that *Actinodaphne* was not monophyletic, but paraphyletic with *Litsea* and *Lindera.* Rohwer [28] and van der Werff [7] also supported that *Actinodaphne* was phylogenetically closer to *Litsea* than other genera in terms of wood and bark anatomical characteristics. Li and Christophel [38], using morphological and leaf cuticle characters, distinguished the difference among *Litsea*, *Lindera*, *Neolitsea* and *Actinodaphne*, and found that *Actinodaphne* has a fasciculate pseudo-umbel inflorescence which differs from *Litsea* because of bract marks at the base of inflorescence. Li et al. [35] obtained a phylogeny of this genus based on 18 species of the Laureae according to ITS and ETS sequences, and found that *Actinodaphne* was closely related to the genus *Neolitsea.* In a subsequent phylogenetic study, Li et al. [39] expanded their taxon samplings containing 29 species of *Neolitsea*, 6 species of *Actinodaphne* and 5 species from the tribes Laureae as outgroups, and they revealed that *Actinodaphne* and *Neolitsea* were sister groups. Further phylogenetic research conducted by Fijridiyanto and Murakami [40], employing the *rpb*2 nuclear gene, and by Liu et al. [41] using the gene fragments of *rbc*L, *mat*K, *trn*H*-psb*A and ITS also found that the *Actinodaphne* and *Neolitsea* were closely related, and they made the point that *Neolitsea*, *Lindera* and *Litsea* were representative genera of the tribe Laureae. According to morphological characteristics, *Actinodaphne* and *Neolitsea* have the same inflorescence type and lack of vegetative terminal buds on the main axis. The main distinguishing features of the two genera are the three cardinal flowers of *Actinodaphne* and the two cardinal flowers of *Neolitsea*, as well as the exfoliated imbricate involucres of *Actinodaphne* and the usually persistent intersecting involucres of *Neolitsea* [28]. In conclusion, the molecular phylogenetic studies above illuminated that *Actinodaphne* was closely related to the representative genera of Laureae: *Neolitsea*, *Lindera* and *Litsea*, and the placement of this genus into the tribe Laureae was well-supported.

The systematic position of *Sassafras* has always been unstable and controversial. *Sassafras* was also part of the Core Lauraceae group and showed different degrees of relatedness with the Laureae and Cinnamoneae on these trees, but they failed to confirm its systematic position. Based on the morphological characteristics of inflorescence without involucre and fruit basal part covered by cupule, *Sassafras* was classified into tribe Cinnamomeae with *Cinnamommum* by Kostermans [3]. In contrast, van der Werff and Richter [2] placed *Sassafras* into the tribe Laureae with the genera *Litsea* and *Lindera* according to the racemose inflorescence surrounded by involucres and the growth ring structure. Kostermans [3] described the flowers of *Sassafras* as short racemes (pseudo-umbals). However, Werff and Richter [2] pointed out that most tribe Laureae species belonged to racemose, and the inflorescence axis was shortened (extremely shortened) and then inflorescence appeared umbellate. In the above two taxonomic systems, both inflorescence type and the presence/absence of involucre of *Sassafras* were emphasized and we consider that the inflorescence type of *Sassafras* is the most discriminating character to determine its systematic placement.

Besides this, whether *Sassafras* has involucre or not is another controversy of its classification. Rohde et al. [9] suggested that most Laureae species have pseudo-umbel axillary inflorescences surrounded by alternate bracts, and the bracts still exist after flowering. However, *Sassafras* produces spikes from spirally arranged bud scales and axils of transitional leaves in early spring germination. It remains to be further studied whether the formation of inflorescence is related to the evolution from spirally inserted involucre to reciprocal involucre.

The phylogenetic studies of the Core Lauraceae group that were undertaken by Li et al. [31] and Nie et al. [36] clearly supported the placement of *Sassafras* into the Cinnamoneae. Furthermore, they showed that the Cinnamoneae and Laureae were sibling groups. In the phylogenetic tree constructed by Rohde et al. [9] based on ITS and chloroplast gene spacers *psb*A*-trn*H and *trn*G*-trn*S, *Cinnamomum* was not monophyletic, but was divided into three groups. Based on the ITS data, *Sassafras* appeared as sister to the lineage composed of paleo-tropical *Cinnamomum* sect. *Cinnamomum* group and the tribe Laureae, while in the *psb*A*-trn*H and *trn*G*-trn*S system trees, it was a sister group with the paleo-tropical *Cinnamomum* sect. *Camphora.* Altogether, based on various DNA sequences analyses, *Sassafras* has a sister relationship with *Cinnamomum*, a core genus of the Cinnamoneae, which provided further molecular evidence for the placement of *Sassafras* in this tribe.

Above all, we proposed that the key point of above controversies is the inflorescence type (raceme, or botryoid and capitellate inflorescences composed of raceme inflorescences) of *Sassafras*, and the closely related evolution relationship between this undetermined inflorescence type and pseudo-umbel inflorescence, cyme panicle inflorescence (additionally: in view of the controversy of *Sassafras*, we have investigated its inflorescence type and re-built the phylogenetic tree using the whole chloroplast genomes of *Sassafras* and its related genera in Lauraceae, and the results are ready to publish).

## 3. The Main Controversy over Perseeae and Cinnamoneae Tribes

Van der Werff and Richter [2] dismantled the Tribe Cinnamoneae established by Kostermans [3] and placed its representative genus *Cinnamomum* in the Perseeae. Several studies concerning systematic relationship [9,42] indicated that whether *Cinnamomum* be classified into the Perseeae [2] or form the Cinnamoneae [3] with *Ocotea* and *Nectandra* will still become the key point in the phylogeny of Lauraceae. In terms of chloroplast gene *trn*K, Rohwer and Rudolph [29] supported that *Cinnamomum* should be one member of the Cinnamoneae with *Ocotea* and *Nectandra*. Rohwer et al. [43] acquired a similar conclusion from phylogenetic analysis of ITS sequence. In view of ITS, *trn*L-*trn*F, *rpl*16 and *psb*A-*trn*H sequences, Nie et al. [36] found a clade including *Cinnamomum*, *Ocotea*, *Aiouea* and *Umbellularia*, which was located between the Laureae clade and the Perseeae clade in phylogeny tree. Song et al. [12] combined the sequences data from *rbc*L, *mat*K, *trn*L*-trn*F, *psb*A*-trn*H, *ndh*F, ITS and *LEAFY* introns to construct a phylogenetic tree containing 23 species of *Persea*, and the results also reflected that *Cinnamomum* was not part of Perseeae. Huang et al. [42] suggested that the *Cinnamomum* should be included in the Cinnamoneae with *Aiouea*, *Ocotea* and several other genera according to the phylogenetic analysis (including 94 species of *Cinnamomum*) of ITS, *LEAFY* and *RPB2* sequences. In general, these researches rather supported that *Cinnamomum* and *Nectandra* should be removed from the Tribe Perseeae and, particularly, that *Cinnamomum* should be classified into the Tribe Cinnamoneae.

## 4. The Systematic Positions of Genera *Neocinnamomum*, *Caryodaphnopsis* and *Cassytha*

Most phylogenetic studies of Lauraceae strongly supported six to seven lineages: Cryptocaryeae, *Cassytha*, *Neocinnamomum*, *Caryodaphnopsis*, Perseeae, Cinnamoneae and Laureae [1,29,44]. Among them, Cryptocaryeae, Perseeae, Cinnamoneae and Laureae are tribes, similar to Kostermans’ [3] classification. Within this phylogenetic framework, the placement of *Caryodaphnopsis*, *Cassytha* and *Neocinnamomum* has not been clearly resolved.

*Neocinnamomum* was originally established by Liu [45] relied on its four-locular anthers with collateral pollen sacs. Kostermans [46] believed that *Neocinnamomum* was extremely close to *Cinnamomum* according to compound thyrse (usually strongly reduced to a few- to many-flowered condensed inflorescence), fleshy fruit receptacles and persistent enlarged tepals. However, Richter [47] suggested that *Neocinnamomum* had a relatively isolated systematic position because of wood and bark, which was closely related to *Caryodaphnopsis.* Van der Werff and Richter [2] did not place this genus into any particular tribe. Recent molecular phylogenetic studies supported that *Neocinnamomum* was closed to *Caryodaphnopsis* and *Cassytha* instead of *Neocinnamomum* and *Cinnamomum*. In view of the ITS, *trn*L*-trn*F and *psb*A*-trn*H sequences, Chanderbali et al. [1] found that *Neocinnamomum* formed a clade with *Cassytha* and *Caryodaphnopsis*, which was between the tribes Cryptocaryeae and Laureae, and *Neocinnamomum* became the sister of *Cassytha* within this clade.

In terms of *trn*K intron sequence, Rohwer and Rudolph [29] also supported the close relationship between *Neocinnamomum* and *Caryodaphnopsis*, and indicated that they are located between the Laureae-Cinnamoneae clade and the Cryptocaryeae clade. Wang et al. [48] revealed the close relationship of *Neocinnamomum*, *Caryodaphnopsis* and *Cassytha* based on the combined data of *psb*A*-trn*H, *trn*K and ITS sequences. Li et al. [49] combined *RPB2*, *LEAFY* and ITS sequences to rebuilt a phylogenetic tree including nine species of *Caryodaphnopsis* (three species from tropical Asia and six from the Neotropics). The results supported that *Caryodaphnopsis* is a monophyletic genera, and showed a close relationship with *Caryodaphnopsis*, *Neocinnamomum* and *Cassytha*. However, these three genera cannot be classified into to any currently recognized tribes; insteadly, they are independently located between the Laureae-Cinnamoneae and Cryptocaryeae tribes.

## 5. Application of Chloroplast Genome in the Phylogeny of Lauraceae

The phylogeny of Lauraceae using single or multiple gene regions has been studied for nearly 20 years. Meanwhile, the conflicting gene trees are still lasting, and the results of molecular phylogenies are divergent with traditional morphological classification in many cases [9,29,36,42].

Chanderbali et al. [1] found that the chloroplast genes (*trn*L*-trn*F, *trn*T*-trn*L, *psb*A*-trn*H and *rpl*16) and nuclear DNA 26S were not powerful to resolve the phylogenetic relationships of tribe Perseeae and Laureae, while ITS sequences could offer better discrimination. Rohwer [30] and Li et al. [31] considered that the *mat*K region was not useful for phylogenetic analysis of the family. Huang et al. [42] found that the ‘short sequence’ copy in the *LEAFY* gene showed low resolution of phylogeny. How to select appropriate molecular data has become one of the new challenges in the study on phylogeny of Lauraceae [21,41].

With the advent of next-generation sequencing technology, the improvement of sequence splicing software, and the continuous reduction of sequencing costs, the chloroplast genome was known as ultra-barcode [50,51,52], and it has been widely used in studies on phylogeny and relatedness of Lauraceae. The chloroplast genome is a small and highly conservative genome with moderate nucleic acid mutation rate and it has rich genetic information and good collinearity and high phylogenetic resolution among the groups [53,54].

*Machilusyunnanensis* Lec and *M. balansae* (Airy Shaw) F.N Wei & S.C Tang were two species in Lauraceae to be first reported in their complete chloroplast genome [55]. Up to December 2020, the NCBI [56] has released the complete chloroplast genome sequences of 104 species of Lauraceae. Song et al. [12] used the complete chloroplast genome data of eight species of Lauraceae to reconstruct a phylogenetic tree and revealed the close relationship among genera *Persea*, *Machilus* and *Phoebe*, which is consistent with the conclusion made by Rohwer et al. [43] based on the ITS sequence, as well as by Li et al. [57] based on the ITS and *LEAFY* intron II sequences.

Song et al. [58] also constructed a phylogenetic tree based on the chloroplast genome data of 34 species of Lauraceae, and the results supported that *Actinodophne* was belonged to the Laureae, and *Sassafras*, *Nactandra* and *Cinnamomum* formed a branch which is separated from the Perseeae. These results offered strong support to the notion that *Cinnamomum*, *Nactandra*, *Sassafras* should be put to the Cinnamoneae. The notion that *Caryodaphnopsis*, *Cassytha* and *Neocinnamomum* form an independent group also received strong support.

Zhao et al. [59] sequenced the complete chloroplast genomes of nine species of *Lindera* and constructed a phylogenetic tree containing 32 species of Lauraceae. Results indicated that *Sassafras*, *Cinnamomum*, *Ocotea* and *Nectandra* constitute a *Cinnamomum*-*Nectandra* clade, which was sister to the *Lindera* clade. The latter includes nine species of *Lindera*, *Laurus nobilis* and *Litsea* *glutinosa.* The study also supported the notion that *Sassafras*, *Cinnamomum*, *Ocotea* and *Nectandra* belong to the Cinnamoneae, and the tribe is closely related to the Laureae. Song et al. [44] constructed a phylogenetic tree based on the chloroplast genomes of 101 species of Lauraceae from 42 genera and revealed nine clades of Lauraceae with six tribes (Laureae, Cassytheae, Neocinnamomeae, Caryodaphnopsideae, Cryptocaryeae, Hypodaphnideae). This study also supported the placement of *Actinodaphne*, *Cinnamomum* and *Sassafras* in the Laureae and did not support the establishment of Cinnamoneae. Furthermore, Song et al. [44] added three new tribes, i.e., Cassytheae, Caryodaphnopsideae and Neocinnamomeae, which supported the isolated *Cassytha*, *Caryodaphnopsis* and *Neocinnamomum* group. However, the resolution of Song et al.‘s [44] tribes is low and differ from Kostermans’ [3], van der Werff & Richter’s [2], Chanderbail et al.’s [1] and Rohwer & Rudolph’s [29] classification systems (Table S1).

## 6. Applications of DNA Barcodings in the Phylogenetic Studies of Lauraceae

Considering that the complete chloroplast genome requires high quality DNA and the cost is high when used as a barcode, micro-barcodes like shorter fragments are easier and cheaper to obtain, and their combinations, especially regions derived from the chloroplast genome, have been widely used in phylogenetic studies of Lauraceae [31,58,59]. It has been found that barcodes of the hypervariable regions of chloroplast genes such as *rpl*32*-trn*L, *ndh*F*-rpl*32, *trn*S*-trn*G, *ndh*F*-rpl*32, *pet*A*-psb*J and *ycf*1 can be applied to resolve the systemic relationship among the Laureae, Cinnamoneae and Perseeae [12,43,55,59,60,61,62]. These DNA barcodes are also suitable for the research on the systematic relationships within genera. For example, *Alseodaphne* has been divided into two genera, *Alseodaphnopsis* and *Alseodaphne* [63], while some species of *Alseodaphne* and *Dehaasia* are still nested in each other’s clades in the phylogenetic tree [43,60,63]. DNA barcodes such as *psb*D*-trn*M and *rpl*32*-trn*L can be used to further distinguish these ambiguous relationships between these two genera [43,60,63,64]. Moreover, the boundary between *Machilus* and *Phoebe* is blurred, and there are some controversial species [57,65,66]. It has been found that barcodes such as *rps*8-*rpl*14, *acc*D, *mat*K, *ndh*J, *psb*A*-trn*H, *psb*B*-psb*H, *psb*C*-trn*S, *rpo*B, *rpo*B*-trn*C, *rpo*C1, *trn*D*-trn*T2, *trn*H*-trn*K, *trn*K and *trn*S*-trnf*M can clarify the phylogenetic relationships among these controversial species and make clear division and classification [43,57,66].

## 7. Discussion and Outlook

The research of molecular systematics of Lauraceae has developed from one or several DNA regions to the recent application of chloroplast genome data to reconstruct phylogenetic trees. Bootstrap value of the Lauraceae phylogenetic tree based on the whole chloroplast genome data generally can reach to 100 percentage, and their trees have higher resolution and reliability [58,59,67]. Based on various molecular data, most of the main controversial issues concerning the Lauraceae phylogeny have been basically clarified. In summary, molecular data support the following conclusions: (1) the establishment of tribe Cinnamoneae, and the placement of genera *Sassafras*, *Cinnamomum*, *Ocotea and Nectandra* in this tribe; (2) the arrangement of *Actinodaphne* in the Laureae; (3) the tribes Cinnamoneae and Laureae are closely related; (4) *Caryodaphnopsis*, *Neocinnamomum* and *Cassytha* are closely related and they are independently located between the Laureae-Cinnamoneae and Cryptocaryeae tribes.

Above all, molecular data have basically illuminated the rationality of the establishment of tribal classifications and the placement of controversial genera. However, the inherent issues such as hybridization, gene introgression, polyploidization, stochastic and systematic errors should be considered when using the molecular data as the evidence of phylogeny [68,69,70]. Therefore, the integrated analysis of phenotypic data and molecular data is the proposed approach for the systematic researches [71,72]. Therefore, the relatively consistent results obtained from the Lauraceae molecular systematic research still need to be discussed in the context of phenotypic traits such as inflorescence characters to reveal the evolutionary path of these traits.

Noteworthily, inflorescence traits have always been considered relevant to solve classification problems of Lauraceae [2,3,21,32,35]. The controversy over the placement of *Sassafras* in a tribe is the most representative issue in the study of Lauraceae phylogeny, which reflects the obvious differences in the definition and evolution of inflorescence types by different taxonomists. Firstly, as a typical East Asian-North American discontinuous genus, *Sassafras* only consists of three species (*S. tzumu*, *S. albidum* and *S. randaiense*). Chung et al. [73] discovered that the inflorescence of *S. randaiense* ended with a terminal flower and these flowers were closely clustered around the terminal buds (pseudo-terminal) of cultivation, forming an appearance similar to the inflorescence type of the tribe Laureae, which resulted in *Sassafras* being classified into the tribe Laureae. We also found that the inflorescence of *S. tzumu* were arranged in a highly reduced panicle or raceme-like cyme, which was similar to tribe Laureae (note: we have a lot of supporting data to be issued).

If the inflorescence of *S. albidum* is similar to the other two species, it suggests that the inflorescence of *Sassafras* should be similar to tribe Laureae. However, most of the molecular evidences support that *Sassafras* should belong to Cinnamoneae. The different results may indicate that the inflorescence of *Sassafras* is a transitional inflorescence type between Laureae and Cinnamoneae. The key point of the above controversies is the inflorescence type (raceme, or botryoid and capitellate inflorescences composed of raceme inflorescences) of *Sassafras*, and the closely related evolution relationship between this undetermined inflorescence type and pseudo-umbel inflorescence, cyme panicle inflorescence. It can be summarized as a debate about which two inflorescence types are more closely related to each other, racemose (represented by *Sassafras*), pseudo-umbels (represented by *Lindera* and *Litsea*) or paniculate (represented by *Cinnamomum*).

Secondly, molecular phylogenetics have revealed that the Cinnamoneae is closely related to the Laureae. The close relationship between these two tribes supports the concept of shortening of the inflorescence axis and the evolutionary model of Brachyblast Type, i.e., a model in which the racemose inflorescence axis is extremely shortened and resembles an umbel [74,75]. It also have indicated that it is partly reasonable for van der Werff & Richter [2] to assign *Sassafras* to the Tribe Laureae. The current molecular systematic studies largely and uniformly supported the placement of *Sassafras* in the Cinnamoneae, that is, the racemose types of *Sassafras* are more closely related to the types of paniculate of *Cinnamomum* and *Ocotea.*

Regarding the work of Chung et al. [73], one can conduct targeted observations on the morphological and structural characteristics (including scanning electron microscopy) of the inflorescences of some controversial genera. For example, one can further verify whether there are any simple phenomenons in the paniculate, especially lateral branch cymes of *Cinnamomum* and *Ocotea.* Additionally, one can determine whether the single florl of racemose of *S. tzumu* and *S. albidumare* has residuals of side branching similar to what can be found in *S. randaiense*.

Molecular data support the relocation of *Actinodaphne* from the Cinnamoneae to the Laureae. The *Actinodaphne* inflorescences are unique in morphology and composition. They are solitary or clustered umbels, panicles or racemose. Are the umbels derived from the extreme shortening of racemose similar to that of the genus of *Sassafras*? Are the clustered umbels, panicles or racemose of *Actinodaphne* species related to the head-like or spike-like arrangement of the racemes of *Sassafras*? Is the minimal pattern of the umbel with only one single flower in the involucre, as seen in *Dodecadenia grandiflora*, *Iteadaphne* *caudata*? All these questions are worthy of further research.

Besides, Neotropical Lauraceae is reasonably well-known according to the intensive fieldwork and recent revisions of some genera [8,10]. Dimitrij et al. [8] constructed a ITS- and *psb*A-*trn*H-based phylogenetic tree of 45 *Nectandra* species plus 42 representatives of 18 genera of the core Lauraceae (*Ocotea* complex, Laureae, *Aiouea*, Asian *Cinnamomum* and *Persea* groups). The results showed that *Nectandra* is diphyletic, which is closer to *Pleurothyrium* and the *Ocotea* complex. Trofimov et al. [10] transferred three species previously included in *Aiouea* to Damburneya, and reinstated the genus *Mespilodaphne* based on ITS and *psb*A-*trn*H sequences of 123 species from the *Ocotea* complex. However, there are little researches on the genomes of these genera, in which internal supports in most phylogenies are low. Nevertheless, Asian Lauraceae outside of China is poorly known. There are no generic revisions (except for a few small genera) and there are few reliably identified collections and few DNA samples. Without more DNA samples, little can be discussed on the generic boundaries and relationships of Asian Lauraceae.

The whole genome sequencing has nowadays been completed for *Persea* *americana*, *Cinnamomum* *kanehirae*, *Phoebe* *bournei* and *Litsea* *cubeba* in the Lauraceae [32,76,77,78]. Although there have been some studies on the whole genome of Lauraceae other than the phylogeny, this review here focuses on the systematics of Lauraceae. Give that the whole genome-sequenced species in Lauraceae is still lacking, which cannot provide more favorable evidence for the phylogenetic relationships of Lauraceae.

The thrilling thing is the researchers have found a conservative gene involved in the development of panicles, FUWA, through the whole genome sequencing [32]. Through genome-wide analysis and in-depth research on functional genes related to the evolution, development and formation of different inflorescence types, we believe that it will be possible to reconstruct a better understanding containing the inflorescence evolution life tree of Lauraceae.

## Figures and Tables

**Table 1:** Comparison of the Kostermans’ and van der Werff’s classifications.

Classification System	Taxonomic Character	Tribe Division	Included Genera
Kostermans’ 1957 [3]	Inflorescence traits Cupule structure	Litseeae	*Adenodaphne*, *Laurus*, *Lindera*, *Litsea*, *Neolitsea*
		Perseeae	*Apollonias*, *Bilschmied*, *Dehaasia*, *Endiandra*, *Haxapora*, *Mezilaurus*, *Persea*, *Phoebe*, *Potameia*
		Cinnamoneae	*Actinidaphne*, *Aiouea*, *Aniba*, *Cinnamomum*, *Dicypellium*, *Endlicheria*, *Licaria*, *Ocotea*, *Phyllostemonodaphne*, *Sassafras*, *Systemonodaphne*, *Umbellulari*, *Urbanodendron*
		Cryptocaryeae	*Cryptocarya*, *EusideroxyIon*, *Potoxyton*, *Ravensara*
		Hypodaphnideae	*Hypodaphnis*
		Subfam. Cassythoideae	*Cassytha*
Van der Werff & Richter’s 1996 [2]	Wood and bark anatomical structure Inflorescence traits	Laureae	*Actinodaphne*, *Litsea*, *Lindera*, *Laurus*, *Sassafras*
		Perseae	*Aniba*, *Cinnamomum*, *Dehassia*, *Licaria*, *Nectandra*, *Ocotea*, *Persea*, *Pleurothyrium*, *Phoebe*
		Cryptocaryeae	*Beilschmiedia*, *Cryptocarya*, *Endiandra*, *Potameria*, *Triadodaphne*

## Data Availability

Not applicable.

## Supplementary Materials

The following are available online at https://www.mdpi.com/article/10.3390/biology10050391/s1, Table S1: Development of Lauraceae Systematics and Classification of Tribes.
