# Supplementary material for: Research Progress in Plant Molecular Systematics of Lauraceae
Source: Biology (Basel). 2021 May 1;10(5):391. doi: 10.3390/biology10050391 (PMC8147330; doi:10.3390/biology10050391)
Supplement: Supplementary file 1 [file biology-10-00391-s001.zip › biology-1183630-supplementary.pdf]

Table S1 Development of Lauraceae Systematics and Classification of Tribes

**classification system**	**Taxonomy character**	**Tribe division**	**Included genera**
Kostermans' 1957[3]	Inflorescence traits<br>Cupule structure	Litseeae	*Adenodaphne, Laurus, Lindera, Litsea, Neolitsea*
		Perseeae	*Apollonias, Bilschmied, Dehaasia, Endiandra, Haxapora, Mezilaurus, Persea, Phoebe, Potameia*
		Cinnamoneae	*Actinidaphne, Aiouea, Aniba, Cinnamomum, Dicypellium, Endlicheria, Licaria, Ocotea, Phyllostemonodaphne, Sassafras, Systemonodaphne, Umbellulari, Urbanodendron,*
		Cryptocaryeae	*Cryptocarya, EusideroxyIon, Potoxyton, Ravensara*
		Hypodaphnideae	*Hypodaphnis*
		Subfam. Cassythoideae	*Cassytha*
Van der Werff & Richter's 1996[2]	Wood and bark anatomical structure<br>Inflorescence traits	Laureae	*Actinodaphne, Litsea, Lindera, Laurus, Sassafras*
		Perseae	*Aniba, Cinnamomum, Dehassia, Licaria, Nectandra, Ocotea, Persea, Pleurothyrium, Phoebe*
		Cryptocaryeae	*Beilschmiedia, Cryptocarya, Endiandra, Potameria, Triadodaphne*
Chanderbail et al.'s 2001[1]	*trn*L-*trn*F, *trn*T-*trn*L, *psb*A-*trn*H, *rpl*l6, 26S,ITS	Laureae	*Actinodaphne, Adenodaphne, Cinnadenia, Dodecadenia, Laurus, Lindera, Litsea, Neolitsea, Parasassafras, Sassafras*
		Cinnamomeae	*Aiouea, Aniba, Cinnamomum, Dicypellium, Endlicheria, Kubitzkia, Licaria, Mocinnodaph, Nectandra, Ocotea, Pleurothyrium, Paraia, Rhodostemonodaphne, Umbellularia, Urbanodendron*

		Persea group	*Alseodaphne, Apollonias, Dehaasia, Machilus, Persea, Phoebe,*
		Chlorocardium-Mezilaurus	*Anaueria, Chlorocardium, Mezilaurus, Sextonia*
		Cryptocaryeae	*Aspidostemon, Beilschmiedia, Cryptocarya, Endiandra, Eusideroxylon, Hypodaphnis, Potameia, Potoxylon,*
Rohwer & Rudolph's 2005[23]	*trn*K intron	Laureae	*Actinodaphne, Laurus, Lindera, Sassafras,*
		Cinnamomeae	*Aniba, Cinnamomum, Endlicheria, Licaria, Nectandra, Ocotea, Umbellularia,*
		Persea group	*Alseodaphne, Apollonias, Dehaasia, Persea,*
		Mezilaurus group	*Anaueria, Chlorocardium, Mezilaurus, Sextonia, Williamodendron*
		Cryptocarya group	*Aspidostemon, Beilschmiedia, Cryptocarya, Endiandra, Eusideroxylon, Potameia*
Song et al.'s 2020[26]	Complete chloroplast genome<br>Wood and bark anatomical structure<br>Inflorescence traits	Laureae	*Actinodaphne, Aiouea, Alseodaphne, Alseodaphnopsis, Anaueria, Aniba, Apollonias, Chlorocardium, Cinnadenia, Cinnamomum, Damburneya, Dehaasia, Dicypellium, Endlicheria, Iteadaphne, Kubitzkia, Laurus, Licaria, Lindera, Litsea, Machilus, Mezilaurus, Mocinnodaphne, Neolitsea, Nectandra, Nothaphoebe, Ocotea, Paraia, Parasassafras, Persea, Pleurothyrium, Phoebe, Povedadaphne, Rhodostemonodaphne, Sassafras, Sextonia, Sinosassafras, Umbellularia, Urbanodendron, Williamodendron,*
		Cassytheae	*Cassytha*
		Neocinnamomeae	*Neocinnamomum*
		Caryodaphnopsideae	*Caryodaphnopsis*
		Cryptocaryeae	*Aspidostemon, Beilschmiedia, Cryptocarya, Dahlgrenodendron, Endiandra, Eusideroxylon, Potameia, Potoxylon, Ravensara, Sinopora, Syndiclis, Triadodaphne, Yasunia*
		Hypodaphnideae	*Hypodaphnis*
